# The Effect of Silver Nanoparticles on Learning, Memory and Social Interaction in BALB/C Mice

**DOI:** 10.3390/ijerph16010148

**Published:** 2019-01-08

**Authors:** Khaled Greish, Abdulelah Abdullah Alqahtani, Abdulla Falah Alotaibi, Ahmed Mohamed Abdulla, Aysha Tariq Bukelly, Fanar Mohammed Alsobyani, Ghazi Hamad Alharbi, Israa Saeed Alkiyumi, Majed Mutlaq Aldawish, Turki Fahad Alshahrani, Valeria Pittalà, Sebastien Taurin, Amer Kamal

**Affiliations:** 1 Department of Molecular Medicine, College of Medicine and Medical Sciences, Arabian Gulf University, Manama 329, Kingdom of Bahrain; Sebastient@agu.edu.bh; 2 Nanomedicine Research Unite, Princess Al—Jawhara Centre for Molecular Medicine and Inherited Disorder, Arabian Gulf University, Manama 329, Kingdom of Bahrain; 3 Department of Physiology, Arabian Gulf University, Manama 329, Kingdom of Bahrain; Abdulelahq@hotmail.com (A.A.A.); abdulla.f.alotaibi@gmail.com (A.F.A.); a.abdulla1994@gmail.com (A.M.A.); ayshanajem@gmail.com (A.T.B.); fanar.alsobyani@gmail.com (F.M.A.); gzoy@hotmail.com (G.H.A.); dralkiyumi94@gmail.com (I.S.A.); majed.aldawish@gmail.com (M.M.A.); dr.t.alshahrani@gmail.com (T.F.A.); amerha@agu.edu.bh (A.K.); 4 Department of Drug Sciences, University of Catania, 95125 Catania, Italy; vpittala@unict.it

**Keywords:** silver nanoparticles, BALB/C mice, behavioural testing, nanotoxicity, learning, memory

## Abstract

Silver Nanoparticles (AgNPs), an epitome of nanotechnology, appear in everyday products such as water filters, printer ink, toothpaste, food packaging and cosmetics mostly due to their bactericidal properties. Given this high level of public exposure, the safety of AgNPs has never been fully established. The unsafe use of AgNPs could pose a real threat, not only to public health but also to economic growth in many industries. In this paper, we tested the effect of AgNPs on memory, learning, social behaviour and motor function of BALB/C mice. Outcomes of the present study suggested an impairment of these functions in AgNPs treated groups. Overall, obtained data support the evidence that the systemic exposure to AgNPs may result in alteration of the cerebral cognition and warrants further consideration on the impact of the AgNPs on human health with respect to their potential neurotoxicity.

## 1. Introduction

The global nanomaterial market was valued at US$7.3 billion in 2016 and the forecast is that it will reach US$16.8 billion by 2022 [1]. Silver nanoparticles (AgNPs) constituted more than 50% of the global nanomaterial marketed products in 2015 [2]. AgNPs are incorporated into products or materials that are encountered daily such as water filters, fabrics, deodorants, printer ink, toothpaste, food packaging and cosmetics [3,4,5,6]. This degree of public exposure is worrisome, given that the safety of AgNPs has never been fully established and their acute and chronic toxicity remain conflicting [7,8,9]. The unregulated use of AgNPs could be a real risk, not only to public health and environment but also to the economic growth of a number of industries. 

Historically, silver has been widely used for more than five millennia for antiseptic activity [10]. Colloidal silver was extensively used in the last century as an antimicrobial agent before the advent of antibiotics in the 40’s. The intensive use of silver in the form of nanoparticles dates back to the beginning of the new century. Conventionally, AgNPs refers to pure elemental silver particles compromising a nanosized structure that ranges from 1 to 100 nm. AgNPs antifungal and bactericidal properties, make them an attractive industrial material in the composition of consumer goods. The antimicrobial activity of AgNPs is related to silver toxicity. Accumulation of the ion inside the cells alters the enzymatic system and may induce lysis due to osmotic perturbation [11]. However, when formulated as AgNPs, silver acquires different properties such as enhanced chemical reactivity and cell permeation, which renders AgNPs more noxious than silver in its free form [12,13]. The strong oxidative activity of AgNPs [14], the ability to cross the placental barrier [15] and blood-brain barrier (BBB) [16,17] invite comparisons with aluminium. Human exposure to aluminium ions has been linked to serious brain dysfunction such as dialysis encephalopathy [18,19,20,21], Alzheimer’s disease [22,23] and Parkinsonism-dementia [24,25,26]. In addition, AgNPs, have been found to cross the BBB to a greater extent than aluminium NPs [22,23]. AgNPs promote BBB dysfunction, astrocyte swelling and neuronal degeneration [27,28]. The mechanism by which AgNPs alter the BBB integrity was associated to the release of proinflammatory mediators including tumour necrosis factor (TNF)-α, interleukin (IL)-1β and prostaglandin E2 (PGE2) by endothelial cells of the cerebral microvessels [29]. These factors increase the cerebral microvasculature permeability and allow the entry of AgNPs and other molecules into the brain.

The adverse effects of AgNPs exposure on memory and spatial learning have been previously studied in different models and some of them with a major focus on prenatal exposure [30,31]. Recently, Węsierska et al. reported that oral gavage with a dose of 1 mg/kg of AgNPs produced adverse effect on memory and cognitive function [32]. In addition, Antsiferova et al. investigated the effects of the prolonged oral administration of PVP-coated AgNPs at the dosage of 50 μg per day demonstrating impairment of long-term memory [33].

In the current study, we propose to determine the impact of AgNPs brain accumulation on the cognitive functions of mice at a very low dosage (0.1 mg/kg, 2 μg per animal). We show that even a low dosage, the accumulation of AgNPs in the mouse brain affect their memory and social behaviour. 

## 2. Materials and Methods 

### 2.1. Design, Synthesis and Characterization of AgNPs

Silver nanoparticles were prepared as described earlier [34]. Briefly, 1 mM silver nitrate was dissolved in 225 mL deionized water, then heated to 95 °C and followed by adding 10 mL of 1% trisodium citrate upon vigorous stirring. After 7 min, the solution was cooled in ice. AgNPs were consolidated by centrifugation at 27,000× g for 15 min (Beckman J2-MC/JA-20, Beckman Coulter, Pasadena, CA, USA) and washed with deionized water thrice by further centrifugation at 31,000× *g* for 20 min (Beckman L-80/NVT-65, Beckman Coulter, Pasadena, CA, USA). Particle size and concentration were measured by dynamic light scattering using a Malvern ZEN3600 Zetasizer Nano series (Malvern Instruments Inc., Westborough, MA, USA). To ensure sterility, AgNPs were filtered through a 0.4 μm pore size filter.

### 2.2. In Vivo Studies

Adult male BALB/C mice, 8–10 week-old and weight 20–25 g, were used in this experiment. The animals had free access to water and fed ad libitum. All the experimental procedures were following the rules and regulations applied by the Research Ethics and Animal Care of the Arabian Gulf University/College of Medicine and Medical Sciences (E005-PI-10/18). Groups of 10 animals each were treated by intravenous injection (i.v.) in the tail vein of 0.1 mL of AgNPs resuspended in water (2 μg/animal). Animals were treated by either single injection, 2 injections with a one week span, 3 injections over 3 weeks duration and control group with no injection. Behavioural testing followed 3 weeks for all treated groups.

#### 2.2.1. Morris Water Maze Test (MWM)

The Morris-water maze was used to test the spatial memory and learning as described earlier [27]. The maze was a circular swimming pool of 140 cm diameter associated with camera tracking computerized system (Stoelting Co., Wood Dall, IL, USA). The maze was divided by the computer program into four quadrants by imaginary lines. The water filling the pool until about its half height was maintained at a temperature of 26–28 °C. Each animal was trained to locate a hidden submerged platform (2 cm below water level). These training sessions were composed of four trials on the first day. Each trial started by releasing the mice from predetermined different quadrants of the pool. The time and distance travelled to reach the hidden platform were calculated by using the video camera tracking system. The experimenter positioned every animal failing to locate the escape platform during the two minutes of the trial time on the platform and left there for 20 s to reorient. Two days later, three test trials for each animal was performed by following the same procedure as described above for training trials of the first day. Immediately after the test trials, a last probe trial was performed during which the platform was removed from the pool and the animals were given 120 s of swimming searching for the escape platform. The percentage of time the animal was spending in each quadrant of the pool was recorded. The animal spending more than 25% of the searching time in the quadrant that contained the platform was considered as learned and remembered the task.

#### 2.2.2. Three-Chambers Social Apparatus Test

Social interaction was tested using three-chambers social apparatus, which is a Plexiglas box (20 × 40 × 22 cm) divided into three compartments with an opening in the middle compartment serving as the entrance of the experimental animal. On both sides of the middle compartment, the other two compartments contain circular wire cage of 9 cm diameter and 11 cm height. The wires were spaced in a way that they provided visual and sensory contacts (like smell) with the encaged animal. The experiment was composed of two sessions. In session 1, an animal (stranger 1) was put in the cage in one of the two-side chambers and the tested (experimental) animal was placed in the middle chamber. The total times spent by the experimental animal in the chamber containing an animal (stranger 1) and the empty chamber were measured by using a video camera. Normal sociability was defined when the experimental animal spent more time in the chamber containing an animal than in the empty chamber. Session 2 measured the social novelty. A new animal (stranger 2) was put on the other chamber which was empty in session 1. Social novelty was defined when the experimental animal spent more time in the chamber containing the new animal than in the chamber containing the old (from session 1). The time allocated for the animal to interact in each session was 10 min. 

#### 2.2.3. Rotarod Performance Test

The rotarod performance test was done utilizing mouse rotarod system. This apparatus is testing the motor power, motor learning and balance of the animals. The test started by placing the mouse on a rotating bar at a speed of 4 m/min. The time the animal can stay on this bar before falling was measured. Every animal was given three trials. The maximum time for each trial was 300 s. The average of the performance in the three trials was calculated. All the groups were tested at the same time of the day.

### 2.3. Statistical Analysis

All data are presented as mean ± SEM unless mentioned otherwise. The significance of learning within the group in the water maze was measured by using Analysis of variance (ANOVA test) by comparing first trial values (latency and duration) with those of the last trial obtained after 48 h.

## 3. Results and Discussion

**Figure 1:**
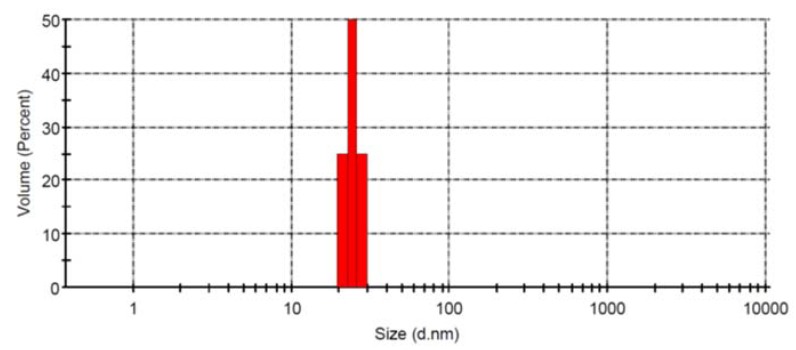
Size distribution of AgNPs. The size distribution of AgNPs was determined using a Malvern Zeta Sizer at a concentration of 1.6 mg/mL in water. Data are expressed as mean ± SEM (*n* = 3).

The present paper aims at elucidating the impact of in vivo treatment with AgNPs on learning, memory and social interaction. AgNPs were prepared by using the well-known chemical reduction method starting from an aqueous solution of AgNO_3_ and using tri-sodium citrate (Na_3_C_6_H_5_O_7_) as reducing and capping agent. The reaction is generally performed at high temperature (>95 °C) to augment the homogeneity in both size and shape of AgNPs. Figure 1 shows the size distribution as measured by DLS. AgNPs, as well as any non-biodegradable molecule exceeding 7 nm, are unable to be excreted by the kidneys [35]. Thus, metallic nanoparticles such as silver will be retained in the body and, as previously studied, will concentrate mainly in organs rich with reticuloendothelial cells such as the liver and spleen [36,37]. The effect on the brain would mainly relate to the uptake by the endothelial cells that make up the BBB. According to previous reports, AgNPs can cross the BBB [16,17,29]. For this purpose, we proceeded with the behavioural testing to examine whether AgNPs would affect important cognitive functions in animal upon reaching the circulatory system.

**Figure 2:**
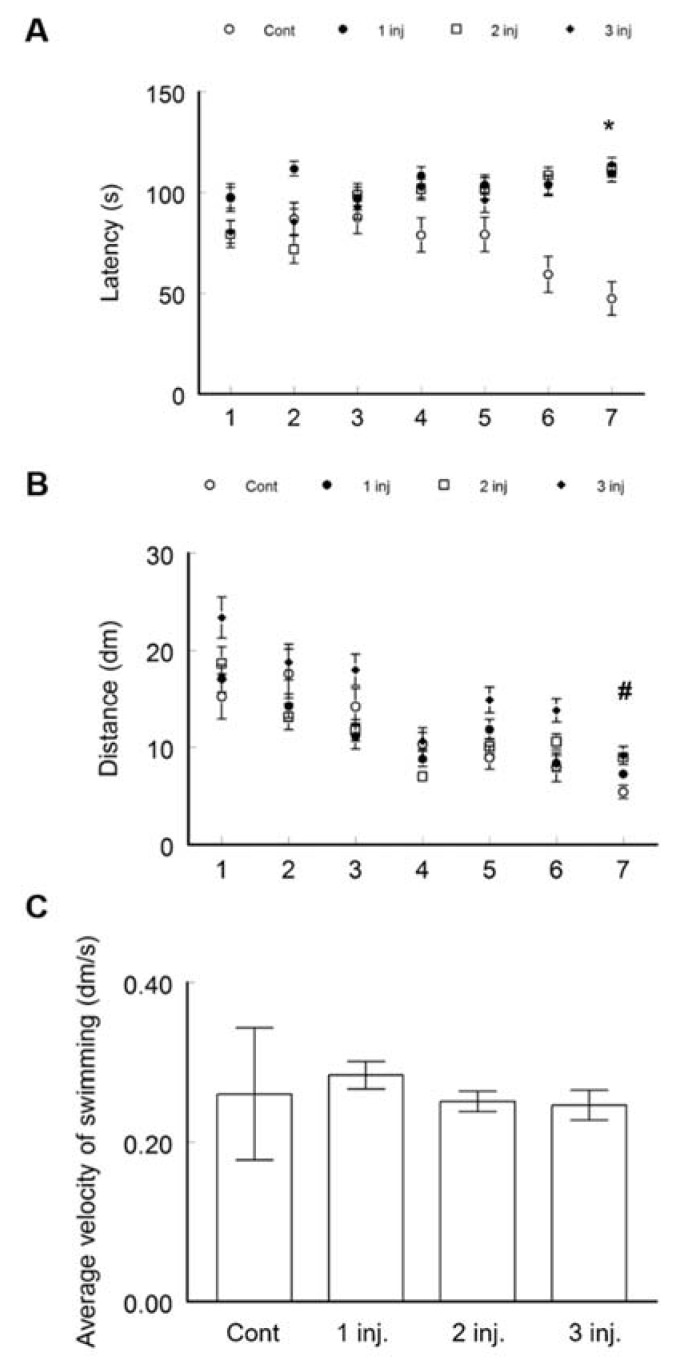
Spatial learning and memory assessed on BALB/C mice using a MWM. Mice were divided into four groups: a control group with no treatment, single i.v. injection in the tail vein of 0.1 mL AgNPs, two IV injections with a one week span and three i.v. injection over 3 weeks duration. (**A**) Latency to reach the hidden platform in the pool. * *p* < 0.05 when comparing the AgNP treated groups to the control group after 7 trials. (**B**) Distance swam to reach the platform. # *p* < 0.05 when comparing the AgNP treated groups with 2 and 3 injections to the control group after 7 trials. (**C**) Average swimming speed. Data are expressed as mean ± SEM (*n* = 3).

Learning and memory were evaluated by MWM (Figure 2). The test is based on sensory cues to swim from a starting point around the perimeter of a small swimming pool to detect a hidden escape platform. The control group showed normal learning behaviour by demonstrating a significant reduction in the latency to reach the submerged platform in the pool between the first trial (113.7 ± 2.9 s) and the last (7th) trial after 48 h (47.3 ± 7.6 s, ANOVA *p* < 0.0001, F; 71.8, F critical; 4.1) (Figure 2A). All the other groups injected with AgNPs failed to show any decrease in the time to reach the submerged platform between the first and the last trial. The distance swam to reach the platform was decreased in the control group between the first trial and the last one (Figure 2B). Conversely, the AgNPs treated animals spent more time in finding the hidden platform and consequently swum more distance. Statistically significant differences were achieved when comparing the latency to reach the platform in the last trial between the control (47.3 ± 8.3 s) and the group receiving one AgNPs injection (109.5 ± 4.2 s, *t*-test *p* < 0.0001, *t* Critical two-tail; 1.989686), two injections (111.1 ± 3.3, *t*-test *p* < 0.0001, *t* Critical two-tail; 2.02) and three injections (114 ± 3.3 s, *t*-test *p* < 0.0001, *t* Critical two-tail; 2.02). However, no statistical differences were recorded in the latency to reach their target between the groups treated with one or two AgNPs injections (*t*-test *p* = 0.8, *t* Critical two-tail; 1.99), or between the ones treated with two or three AgNPs injections (*t*-test, *p* = 0.5, *t* Critical two-tail; 1.98). No statistically significant difference in the swimming speed was detected between all the groups (Figure 2C). This may suggest that the different latency to reach the platform was not due to differences in the speed of swimming but rather to different learning processes among the groups.

**Figure 3:**
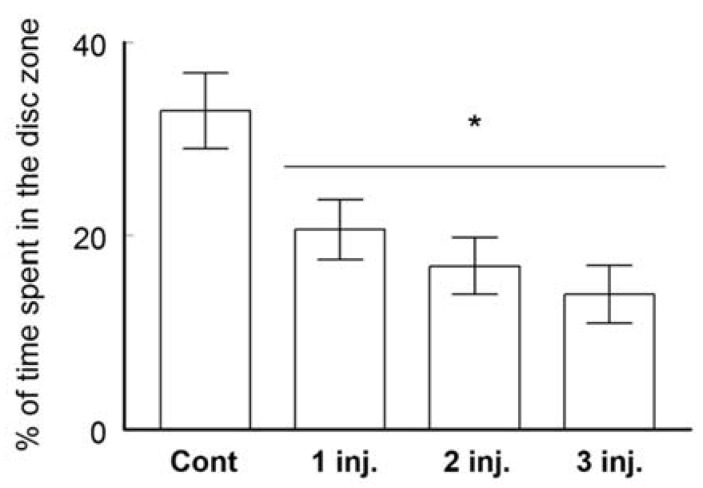
Spatial learning and memory assessed on BALB/C mice using a MWM. Percentage of time spent in the platform (disc) zone by each group of animals. Data are expressed as mean ± SEM (*n* = 3); * *p* < 0.05 when compared to control.

Memory and its recall were measured by the probe test. After removing the platform from the pool, the total time spent by the animals in each quadrant was calculated. (Figure 3). The trial is executed to check if the animals understand the platform location and to observe the strategy followed by animals when they found out the disc is not there. If the animal spent 25% or more of the search time in the previously containing platform quadrant, it is considered having memory of the platform location. The control group spent 32.9% ± 3.9% of the swimming time in the quadrant that contained the platform during the training sessions. This was significantly different from the time spent by the group treated with one injection (20.60 ± 3.1%, *t*-test, *p* < 0.05, *t* Critical two-tail; 1.996564), two injections (16.9 ± 2.9%, *t*-test *p* < 0.005, *t* Critical two-tail; 1.99773) or three injections (14 ± 3% *t*-test *p* < 0.001, *t* Critical two-tail; 1.996008). None of the AgNPs injected animals stayed 25% or more of the swimming time in the platform quadrant. Overall results from the MWM suggested impairment in spatial learning and memory among the groups treated with AgNPs.

**Figure 4:**
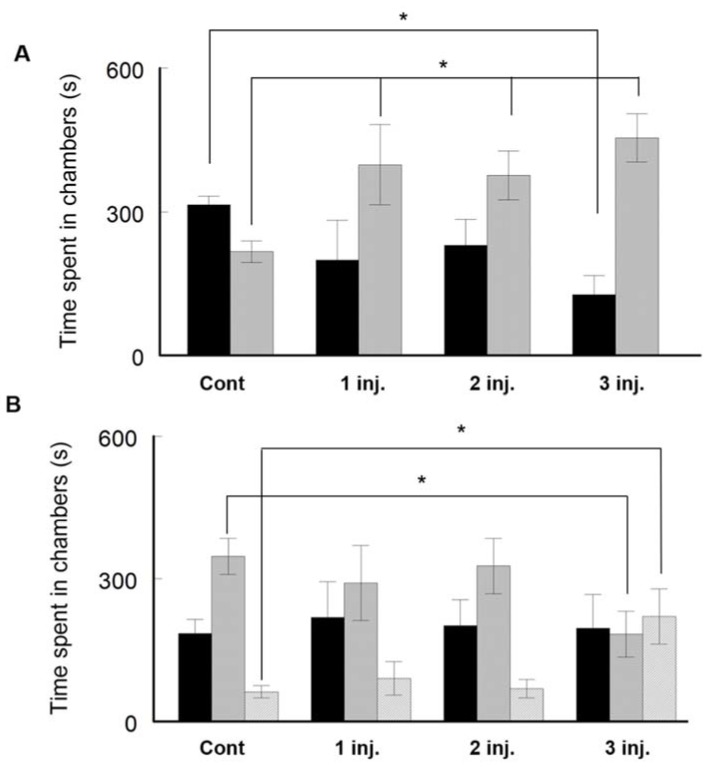
Assessment of social interaction on BALB/C mice by using the three-chambered social test. (**A**) Time spent in an empty chamber or the chamber containing a stranger animal. The black bar shows the time spent in a chamber containing a mouse while the grey bar represents the time spent in an empty chamber. (**B**) Time spent in an empty chamber or the chamber containing a previously explored animal or a newly stranger animal. The black bar represents the time spent in a chamber containing an already encountered mouse; the grey bar illustrates the time spent in a chamber containing a new mouse; while the hatched bar displays the time spent in an empty chamber. Data are expressed as mean ± SEM (*n* = 3); * *p* < 0.05 when compared to control.

The social behaviour of AgNP-treated mice was assessed by using the sociability–social novelty test. The control group spent more time in a chamber containing another animal (stranger 1) (315.4 ± 18 s) when compared to an empty chamber (217 ± 22.4 s, *t*-test; *p* < 0.005, *t* Critical two-tail; 2.14) (Figure 4A) demonstrating a normal social behaviour. On the contrary, the AgNP-treated mice showed an altered social behaviour. The animals of this group preferred to stay significantly more time in the empty chamber than in the chamber containing another animal. Session 2 of this experiment was to test the social novelty of the animals. Normally the animals spend more time in the chamber containing new animal (stranger 2) than in the chamber with the old, already visited animal (stranger 1). The control group animals spent significantly higher time with the new (stranger 2) animal (347 ± 38.1 s) than in the chamber containing the previously explored animal (184.8 ± 29.5 s, *p* < 0.005, *t* Critical two-tail; 2.14) (Figure 4B). The behaviour was different in all the AgNP-treated groups (Figure 4B). Following the 3^rd^ injection, mice spent less time to investigate a new animal (stranger 2) (183.4 ± 48 s) and spent more time in an empty chamber (220.8 ± 48 s) when compared to control animals (62.2 ± 13 s) (Figure 4B). This finding suggests that the treated groups demonstrated abnormal sociability and social novelty behaviours.

**Figure 5:**
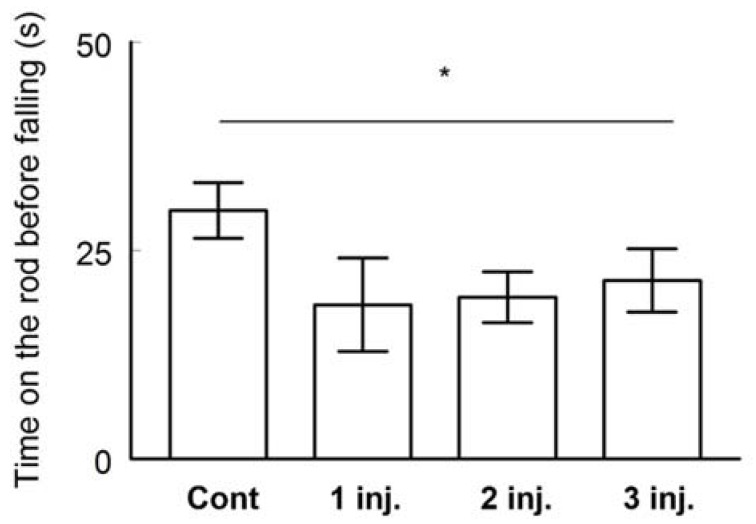
Motor coordination and learning assessed on BALB/C mice by the rotarod test. The time spent on the rod before falling was measured for the four different groups. Data are expressed as mean ± SEM (*n* = 3). * indicates significant difference (*p* < 0.05) between the control group and all the other administered groups. No significant differences were recorded between the three different AgNPs injected groups.

Motor coordination and learning were tested by the rotarod test. The animals able to stay more time on a rotating rod before falling are those considered having good motor coordination and motor learning. The control group spent significantly more time on the rotating rod turning at a speed of 4 m/min before falling (29.78 s). The performance of the control group was significantly better than the AgNPs–injected animals with one (18.49 ± 5.59 s, *t*-test, *p* < 0.05, *t* Critical; 1.770933), two (19.4 ±3.05, *t*-test, *p* < 0.05, *t* Critical two-tail; 2.068658) or three doses of AgNPs (21.39 ±3.78, *t*-test, *p* < 0.05, *t* Critical; 1.734064) (Figure 5). These results indicated lower motor coordination and learning in AgNPs–treated animals when compared to the control.

## 4. Conclusions

Multiple commercial products incorporate in their formulations AgNPs due to their bactericides properties. However, previous studies in animals have demonstrated the ability of these AgNPs to alter the integrity of BBB and promote neurotoxicity following accumulation of silver within the brain. In the present paper, we explored the effects of AgNPs on memory, learning, social behaviour and motor functions. Following injection of AgNPs s (1 to 3 times per experiment, each group), we found a reduction in social interaction and exploratory activity along with impairment in memory, learning and motor functions for all the treated groups. These data presented compelling evidence that the systemic exposure to AgNPs can result in alteration of the cerebral cognition and warrants consideration on the impact of the AgNPs on human health with respect to their neurotoxicity.
